# Latest Advances in Human Brain Dynamics

**DOI:** 10.3390/brainsci11111476

**Published:** 2021-11-08

**Authors:** Stavros I. Dimitriadis

**Affiliations:** 1 Integrative Neuroimaging Lab, 55133 Thessaloniki, Greece; stidimitriadis@gmail.com or DimitriadisS@cardiff.ac.uk; 2 Cardiff University Brain Research Imaging Centre (CUBRIC), School of Psychology, College of Biomedical and Life Sciences, Cardiff University, Cardiff CF24 4HQ, UK; 3 Neuroinformatics Group, Cardiff University Brain Research Imaging Centre (CUBRIC), School of Psychology, College of Biomedical and Life Sciences, Cardiff CF24 4HQ, UK; 4 Division of Psychological Medicine and Clinical Neurosciences, School of Medicine, College of Biomedical and Life Sciences, Cardiff University, Cardiff CF24 4HQ, UK; 5 Neuroscience and Mental Health Research Institute, School of Medicine, College of Biomedical and Life Sciences, Cardiff University, Cardiff CF24 4HQ, UK; 6 MRC Centre for Neuropsychiatric Genetics and Genomics, School of Medicine, College of Biomedical and Life Sciences, Cardiff University, Cardiff CF24 4HQ, UK

It is paramount for every neuroscientist to understand the nature of emerging technologies and approaches in investigating functional brain dynamics. How do all these approaches complement each other? Recognition of the predictive and functional implications of human brain signal dynamics across tasks, cognitive domains, developmental and clinical groups is the first step towards understanding how the brain functions or malfunctions. From a methodological perspective, scientists should be open to novel advanced signal processing techniques (image processing, time-series analysis, etc.), to artificial intelligence algorithms and each subset (machine learning, deep learning), to biologically inspired neural models, and to the association of brain signal dynamics with genes. The investigation of brain signals demands the recognition of all the factors mentioned above that computationally and neurophysiologically constrain human brain dynamics.

Developmental dyslexia is a cognitive linguistic disorder. The study by Dushanova et al. [1] attempted to distinguish subtypes of developmental dyslexia based on the association of speech envelope and electroencephalography (EEG) frequency entrainment adopting a word/pseudoword auditory discrimination experimental paradigm. The main findings of this study revealed aberrant frequency-dependent entrainments of distinct brain areas related to the task differentiating phonological dyslexia from visual dyslexia. In a study by Chikara and Ko [2], authors investigated inter and intra-subject variability of human inhibitory control via a stop signal task under a realistic environmental scenario. The combination of EEG signal processing with machine learning revealed essential brain areas and frequencies linked to the task that further explained intra and inter-subject variability of the inhibitory control. These findings could improve the psychopathology of attention deficit hyperactivity disorder (ADHD), obsessive-compulsive disorder (OCD), schizophrenia, and suicidality.

Kotiuchyi et al. [3] attempt for the very first time to assess the information dynamics of EEG source time series via time-lagged source interactions and instantaneous mixing effects. The authors combined common spatial patterns (CSP) technique, vector autoregressive (VAR) models, and independent component analysis (ICA). They applied this novel framework in a dataset of epileptic patients to describe the changes of brain information dynamics in both sensor/source levels due to epileptic seizures. In the case of generalized seizures, similar information–theoretical measures were estimated in both scalp and source signals while a different level was detected between the two types of signals in focal seizures.

Deep convolutional neural networks (CNNs) and human visual systems have demonstrated common invariances identified or classified after rotation, scaling, and translation transformations. Thus, representation invariance plays a typical role in CNN and human visual processing information under complicated image-based tasks. To investigate this relationship between CNNs and the human visual system, Cui et al. [4] explored the representation invariances of CNNs and the ventral visual stream by comparing features from the layers of CNNs and the prediction performance of visual encoding models. This novel study untangled the importance of invariant representation of computer vision and the deeper conception of the representation invariant mechanism of the human visual information processing.

Genetic neuroimaging is a relatively new scientific field that focuses on integrating single-modal or multi-modal neuroimaging data with genomics with the primary aim to explore the mechanisms that substrate neurodegenerative and neuropsychiatric disorders and brain phenotypes. Rebelo et al. [5] investigated novel quantitative trait loci (QTLs) for brain wave relative power (R.P.) by combining EEG resting-state recordings across five frequency bands (δ, θ, α, β1, and β2) and genome-wide data in a cohort of patients with late-onset Alzheimer’s disease (LOAD), individuals with mild cognitive impairment, and controls. This study revealed two interesting findings. First, *CLEC16A* (C-type lectin domain family 16), with a variant at this locus, was associated with brain wave biology, enhancing the immune system’s significant role in brain function. Furthermore, independent association signals comprise brain expression quantitative loci (eQTLs) in specific loci associated with pathologies and neurological traits.

The combination of noninvasive medical neuroimaging with brain connectivity and especially network neuroscience has yielded many discoveries about how brain topology is altered in various brain disorders and diseases. Lazarou et al. [6] investigated for the very first time how network topology could be altered in preclinical stages of Alzheimer’s disease (AD) and namely in subjects with subjective cognitive decline (SCD) in combination with subjects with healthy controls (HC), with mild cognitive impairment (MCI) and with AD. The authors analyzed resting-state EEG recordings acquired from a 256 High Density-EEG system. Following a network analysis on the sensor level, they reported a disrupted network topology in parietal areas for subjects in preclinical stages of AD, opening new ways of designing biomarkers across the continuum of AD.

Brain activity and connectivity encapsulate important and complementary features related to human brain dynamics that can further feed a machine learning scheme. A novel study by Provenzano et al. [7] attempts to identify the critical brain areas in which their brain pattern can differentiate subjects with Gulf War Illness (GWI) and chronic fatigue syndrome (CFS). Both disorders share similar symptomatology that involves fatigue, chronic pain, and exertional exhaustion after exercise. Physicians have believed that both symptoms are psychosomatic disorders, and until now, no etiology has been revealed. The authors of this novel study scanned many subjects with both symptoms using fMRI modality while the subjects responded to a 0 (baseline) and a 2-back working memory task before and after exercise. Adopting a variety of classifiers with features linked to the brain activity of parcellated brain areas, authors succeeded in differentiating both symptoms based on multi-regional fMRI brain activity with an average performance of 75%.

Patients with systemic lupus erythematosus (SLE) frequently show symptoms of central nervous system (CNS) involvement, termed neuropsychiatric SLE (NPSLE). The CNS manifestations of SLE are diverse and have a broad spectrum of severity and prognostic implications. NPSLE encompasses a variety of neurological and psychiatric signs and symptoms that are often hard to distinguish from SLE-unrelated events. The nervous system is one of the major organs affected in patients with systemic lupus erythematosus (SLE). Research interest in neuropsychiatric SLE (NPSLE) has seen major growth during the past 5 years, which is largely attributable to the understanding that NPSLE develops along unique pathogenetic pathways compared with other SLE manifestations. Neuropsychiatric (NP) events occur in the majority of patients with SLE and predominantly affect the CNS additionally to the peripheral and autonomic systems. NPSLE represents approximately 30% of all NP events in SLE patients and occur more frequently by the SLE onset. It seems that the pathogenesis of NPSLE includes two disease pathways, an ischaemic or neuroinflammatory. However, current research on the pathophysiological substrate of NPSLE manifestations, including neuroimaging, is minimal. The study by Simos et al. [8] is the first one in the literature that investigates how subject-specific whole-brain functional connectivity networks derived from resting-state fMRI recordings are affected in subjects with NPSLE compared to age-matched healthy controls (HC). A network neuroscience approach combined with machine learning revealed important network features linked to 11 brain regions associated with a 73.5% classification performance (NPSLE vs. HC). These findings are supported by earlier work regarding hemodynamic disturbances in these brain regions in NPSLE subjects and associations of these metrics with visuomotor performance and flexibility scores acquired by the NPSLE subjects.

Brain neuroimaging charts of human lifespan are an essential step for quantifying and standardization of individual variation and characterizing deviations from age-dependent normal trends. A unique functional neuroimaging study by Dimitriadis et al. [9] presented for the very first time how intrinsic functional connectivity patterns between specific brain areas are altered across the lifespan (8–60 years). They analyzed resting-state recordings acquired with magnetoencephalography (MEG) under the brain connectivity framework by dynamically adopting various brain connectivity estimators. The estimators were dedicated to within-frequencies and between-frequencies coupling (cross-frequency coupling) in both amplitude and phase domain. Multi-class support vector machines achieved 89% correct participants according to their chronological age using dynamic functional connectivity indices. A flexibility index (FI) defined by the temporal evolution of the dominant intrinsic coupling mode using a proposed dominant coupling mode (DoCM) model showed an inverse U-shaped curve across healthy individuals. Furthermore, the authors showed a reduction of this FI in children with reading difficulties and a cohort with mild traumatic brain injury compared to the age-matched healthy individuals. Furthermore, measures of FI were repeatable in a separate test–retest study and were robust between recordings derived from different MEG systems.

Magnetic resonance imaging (MRI) is a medical imaging technique used in radiology to form pictures of the anatomy and the physiological processes of the body. MRI scanners use strong magnetic fields, magnetic field gradients, and radio waves to generate images of the organs in the body. Diffusion-weighted magnetic resonance imaging (DWI or DW-MRI) uses specific MRI sequences and software that generates images from the resulting data using the diffusion of water molecules to generate contrast in MR images. A special kind of DWI, diffusion tensor imaging (DTI), has been used extensively to map the brain’s white matter tractography. Another interesting study of this special issue investigates the potential correlations of the duration of breastfeeding with major tracts in 4- to 8-year-old children using diffusion tensor imaging technique. Bauer et al. [10] found a significant correlation between fractional anisotropic scores in white matter tracts in the left hemisphere, including pathways known to be functionally relevant for reading and language development.

A methodological study by Wang et al. [11] proposed a novel superpixel segmentation algorithm by integrating texture features that further improved simple linear iterative clustering (SLIC). Segmentation of MRI images is of paramount importance, and it is an open task that deserves further exploration. However, non-uniform grey distribution and blurred edges often bias the superpixel segmentation. This study proposed a combination of a 3D histogram reconstruction, a local tri-directional pattern descriptor for feature extraction, and a novel clustering algorithm. The results showed that the proposed updated superpixel segmentation method outperformed state-of-the-art methods in whole-brain parcellations, especially in fuzzy boundaries and fuzzy brain areas.

Another study by Ryu and Park [12] investigated the relationship between the structural characteristics of the left arcuate fasciculus (AF) reconstructed using diffusion tensor images (DTI) to the type of fluent aphasia according to hemorrhage lesions in patients with fluent aphasia following intracranial hemorrhage (ICH). They analyzed the dataset of five subjects with fluent aphasia following ICH, assessing the patient’s language function with the Korean version of Western Aphasia Battery. All patients showed neural tract injury estimated with DTI parameters, while individualized structural properties of the AF in the left hemisphere were associated with various types of fluent aphasia.

An important aspect of computational neuroscience is designing models that improve connectivity maps, which further allows the realization of the long-standing goal of understanding the interplay between structural topology and brain dynamics in fine-scaled cortical networks. These computational neuroscience models work with the activity of spiking neurons and attempt to understand whether this complex cortical wiring is due to evolutionary processes that optimize region-to-region communication or some higher cognitive function. For example, the study by Pena et al. [13] adopted an information–theoretical approach to explore the activity propagation in spiking neural networks in conjunction with a hierarchical modular topology. They finally observed that the optimized pairwise information propagation emerges due to the increase of the global synaptic strength parameter or the number of modules in the network while the network size remains constant. Moreover, the information propagation of activity between adjacent modules is enhanced with the number of modules until a plateau, showing an optimal relationship between synaptic strength and modularity for population information flow.

Moreover, the increase of synaptic strength and the number of modules are associated with the increase of autocorrelations between individual neurons and the increase of cross-correlations among pairs of neurons. The second effect is associated with better information propagation in the network. Therefore, this study suggests ways of how synaptic strength and topological properties are linked to information transmission.

Electron microscopy (EM) is a technique for obtaining high-resolution images of biological and non-biological specimens. It is mainly used in biomedical research to investigate the detailed structure of tissues, cells, organelles, and macromolecular complexes. With the extensive use of EM and the demand for neuron circuit reconstruction, the scale of reconstructed data is growing exponentially. This action brings the central challenge: how effectively neuroscientists can manage large-scale data to retrieve valuable information more easily. A solution to this problem is described in the study by Yuan et al. [14], who developed a data management module equipped with a storage and a retrieval module on the server-side and an image cache module on the client-side. Hadoop and HBase are introduced on the server-side to resolve massive data storage and retrieval, while on the client-side, a three-level image cache module is designed to reduce latency when acquiring data. The proposed client–server architecture has been tested successfully, showing excellent real-time performance when handling large-scale data.

Transcranial electrical stimulation (tES) is a noninvasive brain stimulation technique that applies a weak current and electrical current through the cortex of the brain in order to alter brain function. In recent years, tES has been employed in investigating the neural processes involved in human behavior. An essential application of tES is to study central auditory processes (CAP) via analyzing the related phenomena that include sound localization, auditory pattern recognition, and auditory discrimination. CAP is the perceptual processing of auditory information in the central auditory nervous system (CANS) and the neurobiological activity that underlies this processing giving rise to electrophysiologic auditory potentials. Knowledge of the neuroanatomy and physiology of the CANS is essential for a better understanding of the underlying processes and deficits. This knowledge can further improve our interpretation of the positive or negative impact of intervention with tES. However, the application of tES in the field of auditory processes is minimal, and Wang and his colleagues [15] reviewed the effect of tES on behavior, auditory and cognitive function, summarizing the current physiological effects of tES on the auditory cortex.
